# Hyponatremia as a Prognostic Marker in Cirrhosis: A Systematic Review of Its Role Beyond the Model for End-Stage Liver Disease in Predicting Mortality

**DOI:** 10.7759/cureus.112069

**Published:** 2026-07-04

**Authors:** Mounika Kotte, Sally M Nashed, Aryan Raj, Vimi Bansal, Sunita Kumawat, Shaimaa Awad, Taha Khalid

**Affiliations:** 1 Internal Medicine, Southern Regional Medical Center, Riverdale, USA; 2 Forensic Medicine and Toxicology, Ain Shams University, Cairo, EGY; 3 Internal Medicine, Shaheed Mohtarma Benazir Bhutto Medical University, Larkana, PAK; 4 Internal Medicine, Maharishi Markandeshwar University, Mullana, IND; 5 Internal Medicine, St. Francis Medical Center, Lynwood, USA; 6 Internal Medicine, Nottingham University Hospitals NHS Trust, Nottingham, GBR; 7 Internal Medicine, Nishtar Medical University, Multan, PAK

**Keywords:** cirrhosis, hyponatremia, liver transplantation, mortality, prognosis, risk stratification, serum sodium

## Abstract

This systematic review evaluates the prognostic significance of hyponatremia in patients with cirrhosis across diverse clinical settings. A comprehensive literature search was conducted across PubMed/MEDLINE, Scopus, and Web of Science for studies published between January 2000 and December 2025, in accordance with the Preferred Reporting Items for Systematic Reviews and Meta-Analyses (PRISMA) guidelines and a structured Population, Intervention, Comparison, and Outcomes (PICO) framework. Observational cohort studies assessing the association between serum sodium levels and mortality outcomes in adult patients with cirrhosis were included. Seven studies met the eligibility criteria, encompassing heterogeneous cohorts, including critically ill patients, transplant waitlist populations, and individuals with advanced disease states such as refractory ascites. Across the included studies, hyponatremia consistently emerged as an independent predictor of mortality, with prognostic significance observed across varying degrees of disease severity and clinical contexts. Notably, serum sodium demonstrated additional risk-stratification value beyond the Model for End-Stage Liver Disease (MELD) score, particularly among patients with lower MELD scores, suggesting its utility in identifying high-risk individuals who may otherwise be underestimated by conventional prognostic models. From a pathophysiological perspective, hyponatremia reflects underlying circulatory and neurohumoral dysfunction, integrating key features of decompensated liver disease into a single clinically measurable parameter. Despite heterogeneity in study design and definitions of hyponatremia, the included studies generally demonstrated moderate methodological quality. Collectively, these findings support the incorporation of serum sodium into prognostic assessment frameworks while emphasizing the need for cautious interpretation and further research focusing on dynamic sodium trends and multidimensional risk-prediction models.

## Introduction and background

Chronic liver disease and cirrhosis remain major contributors to global morbidity and mortality, with disease progression characterized by portal hypertension, systemic circulatory dysfunction, and multiorgan involvement. As patients transition from compensated to decompensated stages, the risk of adverse outcomes increases substantially, necessitating reliable tools for risk stratification and prognostication [1]. The Model for End-Stage Liver Disease (MELD) score has been widely adopted for this purpose, particularly in the setting of liver transplantation, because of its ability to predict short-term mortality using objective laboratory parameters [2]. However, clinical observations have demonstrated that patients with relatively low MELD scores may still experience significant morbidity and early mortality, suggesting that additional factors contribute meaningfully to disease severity and prognosis [3].

Hyponatremia is a common and clinically significant electrolyte disturbance in cirrhosis, typically resulting from impaired free water excretion due to non-osmotic vasopressin release in the setting of effective arterial hypovolemia [4]. It is closely associated with advanced portal hypertension, splanchnic vasodilation, and activation of neurohumoral pathways such as the renin-angiotensin-aldosterone system. Beyond its pathophysiological basis, hyponatremia has been linked to a range of complications, including hepatic encephalopathy, ascites, spontaneous bacterial peritonitis, and hepatorenal syndrome. These associations have generated increasing interest in serum sodium as a potential marker of disease severity and systemic circulatory derangement in cirrhosis [5].

A growing body of observational evidence has evaluated the relationship between serum sodium levels and clinical outcomes in patients with chronic liver disease. Several studies have suggested that hyponatremia independently predicts mortality across diverse clinical settings, including patients awaiting liver transplantation, individuals with refractory ascites, and critically ill patients admitted to intensive care units (ICUs) [6,7]. Furthermore, the incorporation of serum sodium into established prognostic models has been explored as a means of improving predictive accuracy. Nevertheless, the available literature remains heterogeneous with respect to patient populations, definitions of hyponatremia, and outcome measures, which may influence the interpretation and generalizability of findings [8].

The objective of this systematic review is to evaluate the prognostic significance of hyponatremia in adult patients with chronic liver disease and cirrhosis, with particular emphasis on its association with mortality outcomes. By synthesizing evidence from cohort studies and related observational analyses, this review aims to clarify the role of serum sodium as an independent predictor of mortality and to examine its potential contribution to risk stratification across different clinical contexts.

## Review

Materials and methods

Study Design and Reporting Framework

This systematic review was conducted in accordance with the Preferred Reporting Items for Systematic Reviews and Meta-Analyses (PRISMA 2020) guidelines [9]. The methodology was structured around a clearly defined Population, Intervention, Comparison, and Outcomes (PICO) framework [10] to ensure methodological rigor and reproducibility. The population of interest comprised adult patients with liver cirrhosis across diverse clinical settings, including transplant waitlists, ICUs, and advanced stages of disease. The exposure of interest was hyponatremia, defined as reduced serum sodium levels and evaluated as either a categorical or continuous variable. Comparators included patients with normal serum sodium levels or analyses adjusted for established prognostic models such as the MELD score. The primary outcomes included short- and intermediate-term mortality, encompassing in-hospital mortality and survival at three, six, and 12 months.

Data Sources and Search Strategy

A comprehensive literature search was conducted across three major electronic databases: PubMed/MEDLINE, Scopus, and Web of Science. The search strategy was designed to identify studies evaluating the prognostic significance of serum sodium in cirrhosis and was restricted to articles published between January 2000 and December 2025, reflecting the evolution of MELD-based prognostic frameworks and the emergence of Model for End-Stage Liver Disease-Sodium (MELD-Na) concepts. Both Medical Subject Headings (MeSH) and free-text terms were used and combined with Boolean operators to optimize sensitivity and specificity. Key search terms included “cirrhosis,” “liver cirrhosis,” “serum sodium,” “hyponatremia,” “MELD score,” “MELD-Na,” “mortality,” “survival,” and “liver transplantation.” These terms were combined using operators such as AND and OR; for example: (“cirrhosis” OR “liver cirrhosis”) AND (“hyponatremia” OR “serum sodium”) AND (“mortality” OR “survival”) AND (“MELD” OR “liver transplantation”). In addition, the reference lists of included studies were manually screened to identify potentially relevant articles.

Study Selection Process

Studies identified through the search strategy were screened in a stepwise manner. Titles and abstracts were initially reviewed to exclude clearly irrelevant articles, followed by full-text assessment of potentially eligible studies. The selection process adhered to PRISMA principles, with emphasis on transparency and reproducibility. Studies were selected based on their relevance to the predefined research question and their ability to provide clinically meaningful data on the association between serum sodium levels and mortality outcomes in cirrhosis.

Eligibility Criteria

Studies were included if they were observational cohort studies, whether prospective or retrospective, that evaluated adult patients with cirrhosis and examined the association between serum sodium levels or hyponatremia and mortality outcomes. Eligible studies were required to report clinically relevant endpoints such as in-hospital mortality, short-term mortality ranging from three to six months, or longer-term outcomes up to 12 months. Studies that assessed the prognostic value of serum sodium independently or in conjunction with established scoring systems such as MELD were considered particularly relevant, as they aligned with the objectives of this review.

Exclusion criteria included studies involving pediatric populations, case reports, narrative reviews, editorials, and studies lacking clear outcome measures or statistical analysis. Studies that did not evaluate serum sodium as an independent prognostic factor or failed to report mortality-related outcomes were also excluded. This approach ensured the inclusion of studies that were methodologically aligned and directly relevant to the prognostic evaluation of hyponatremia in cirrhosis.

Data Extraction and Synthesis

Data extraction was performed using a standardized approach to ensure consistency across studies. Key variables extracted included study design, sample size, patient population characteristics, definition of hyponatremia, outcomes assessed, and principal findings related to mortality risk. Given the heterogeneity in study populations, definitions of hyponatremia, and outcome measures, a quantitative meta-analysis was not performed. Instead, a structured narrative synthesis was undertaken, focusing on identifying consistent patterns, subgroup-specific findings, and clinically meaningful insights across the included studies.

Quality Assessment

The methodological quality and risk of bias of included studies were assessed using the Newcastle-Ottawa Scale (NOS) for cohort studies [11]. This tool evaluates studies across three domains: selection of participants, comparability of study groups, and assessment of outcomes. Studies were categorized as low, moderate, or high risk of bias based on their overall scores and methodological robustness. This assessment was incorporated into the interpretation of findings to ensure that conclusions were grounded in the quality of the available evidence.

Results

Study Selection Process

**Figure 1:**
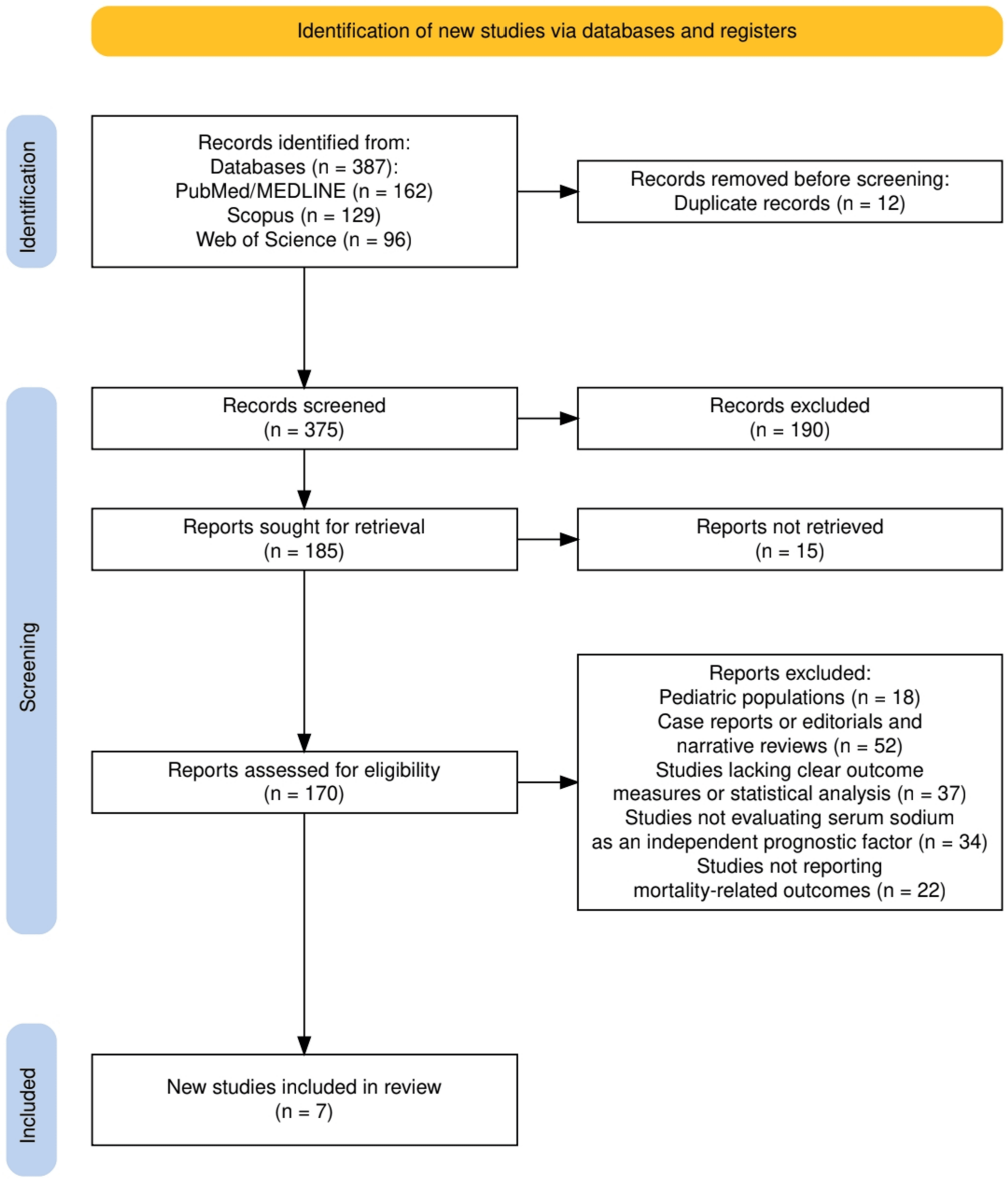
The PRISMA flow diagram depicting the process of the selection of the studies. PRISMA: Preferred Reporting Items for Systematic Reviews and Meta-Analyses

The study selection process is summarized in Figure 1 and follows a structured PRISMA-based approach. A total of 387 records were initially identified through database searching, with 12 duplicates removed prior to screening, leaving 375 records for title and abstract review. Of these, 190 records were excluded based on lack of relevance, and 185 reports were sought for full-text retrieval, of which 15 could not be accessed. A total of 170 full-text articles were assessed for eligibility, and 163 were excluded based on predefined criteria, including studies involving pediatric populations, non-original articles, such as case reports and reviews, studies lacking clear outcome measures or statistical rigor, and those not evaluating serum sodium as an independent prognostic factor or not reporting mortality outcomes. Ultimately, seven studies met the inclusion criteria and were incorporated into the final qualitative synthesis, as illustrated in Figure 1.

Characteristics of the Selected Studies

The included studies, summarized in Table 1, primarily consisted of observational cohort designs, incorporating both prospective and retrospective methodologies across diverse clinical settings. Study populations ranged from critically ill patients admitted to ICUs to individuals on liver transplant waitlists and those with advanced disease manifestations such as refractory ascites. Sample sizes varied moderately, reflecting both single-center and multicenter experiences, and most studies evaluated adult patients with cirrhosis across different stages of disease severity. Definitions and reporting of hyponatremia were not uniform across the included studies. Some studies used categorical thresholds such as Na ≤135 mmol/L, Na <130 mEq/L, Na <126 mEq/L, or ≤125 mmol/L, whereas others assessed serum sodium as a continuous variable. Admission sodium levels, median sodium values, and sodium distributions according to mortality or survival groups were not consistently reported. As a result, direct comparison across mild, moderate, and severe hyponatremia categories was limited. Across studies, mortality outcomes were consistently reported, including in-hospital, short-term, and intermediate-term survival, with several analyses incorporating established prognostic models such as MELD for comparative or adjusted evaluation.

**Table 1:** Summary of observational cohort studies evaluating hyponatremia as a prognostic marker of mortality in patients with cirrhosis across diverse clinical settings. CI: confidence interval; HR: hazard ratio; ICU: intensive care unit; MELD: Model for End-Stage Liver Disease; MELD-Na: Model for End-Stage Liver Disease-Sodium; Na: sodium

Study (Year)	Design	Population	Hyponatremia Definition	Outcome	Key Findings	Notes
Jenq et al. (2010) [12]	Prospective cohort	126 cirrhotic patients admitted to ICU	Na ≤135 mmol/L	In-hospital and 6-month mortality	Hyponatremia independently predicted 6-month mortality (Cox analysis); higher in-hospital mortality observed	ICU/critically ill subgroup; high baseline mortality (65%)
Prohic et al. (2016) [13]	Cohort	115 cirrhotic patients (non-transplant)	Low serum sodium (threshold not explicitly specified)	6-month mortality	Hyponatremia independently predicted mortality in MELD <21; better prognostic accuracy than MELD in this subgroup	Low MELD subgroup; highlights limitation of MELD
Sersté et al. (2012) [14]	Prospective cohort	174 cirrhotic patients with refractory ascites	Severe hyponatremia ≤125 mmol/L	1-year mortality	Severe hyponatremia independently predicted mortality; stronger predictor than MELD-Na	Advanced/decompensated subgroup (refractory ascites)
Moini et al. (2011) [15]	Retrospective cohort	612 cirrhotic patients on transplant waitlist	Na <130 mEq/L (also <135 mEq/L explored)	90- and 180-day mortality	Hyponatremia (<130 mEq/L) independently predicted early mortality alongside MELD	Transplant waitlist cohort; short-term mortality focus
Londoño et al. (2007) [16]	Prospective cohort	308 cirrhotic patients awaiting liver transplantation	Serum sodium (continuous variable)	3- and 12-month mortality	Serum sodium independently predicted mortality alongside MELD; consistent across subgroups	Large transplant cohort; comparison with MELD
Biggins et al. (2005) [17]	Retrospective cohort	513 cirrhotic patients on transplant waitlist	Na <126 mEq/L	3- and 6-month mortality	Severe hyponatremia independently predicted mortality (HR ↑); improved prognostic accuracy of MELD	Landmark study; foundation for MELD-Na
Heuman et al. (2004) [18]	Retrospective cohort (with validation cohort)	507 cirrhotic patients referred for transplantation	Na <135 mEq/L	180-day mortality	Hyponatremia independently predicted early mortality; particularly significant in MELD <21 and validated in separate cohort	Strong methodological design (derivation + validation); low MELD subgroup focus

Risk of Bias Assessment

The methodological quality of the included studies was assessed using the NOS, and the findings are summarized in Table 2. Overall, the studies demonstrated moderate to high methodological quality, with most achieving strong scores in the selection and outcome domains due to well-defined cohorts and clinically relevant, objective endpoints such as mortality. Several studies also incorporated multivariable analyses, enhancing comparability and reducing confounding. However, as all included studies were observational in nature, a degree of residual confounding cannot be excluded. Additionally, variations in population characteristics, definitions of hyponatremia, and study settings introduce potential sources of bias. Despite these limitations, the overall risk of bias was considered low to moderate, supporting the reliability of the synthesized findings while warranting cautious interpretation.

**Table 2:** Risk of bias assessment of included cohort studies using the Newcastle-Ottawa Scale. MELD: Model for End-Stage Liver Disease; Na: sodium; NOS: Newcastle-Ottawa Scale

Study (Year)	Study Design	Suitable RoB Tool	Likely NOS Score (/9)	Overall Risk of Bias	Brief Justification
Jenq et al. (2010) [12]	Prospective cohort	NOS	8/9	Low to moderate	Prospective data collection, clear exposure and mortality outcomes, and multivariable analysis strengthen validity; limited by single-center ICU population and subgroup-specific generalizability.
Prohic et al. (2016) [13]	Cohort	NOS	6/9	Moderate	Relevant prognostic question and short-term follow-up, but smaller sample size, unclear sodium threshold definition, and less detailed adjustment strategy increase risk of bias.
Sersté et al. (2012) [14]	Prospective cohort	NOS	8/9	Low to moderate	Consecutive inclusion, clinically well-defined refractory ascites population, and robust mortality analysis support quality; single-center design and highly selected advanced subgroup limit external validity.
Moini et al. (2011) [15]	Retrospective cohort	NOS	7/9	Moderate	Large transplant waitlist cohort and clinically relevant mortality endpoints are strengths; retrospective design and potential selection/confounding issues reduce internal validity.
Londoño et al. (2007) [16]	Prospective cohort	NOS	8/9	Low to moderate	Well-characterized transplant cohort, multivariable analysis, and clearly defined outcomes strengthen quality; restricted transplant population and absence of clear incremental benefit over MELD slightly limit applicability.
Biggins et al. (2005) [17]	Retrospective cohort	NOS	8/9	Low to moderate	Strong cohort size, explicit sodium threshold, hazard ratios, and clinically relevant mortality prediction are major strengths; retrospective single-center design remains a limitation.
Heuman et al. (2004) [18]	Retrospective cohort with validation cohort	NOS	8/9	Low to moderate	Derivation and validation cohorts substantially strengthen methodological rigor; retrospective design and heavily male veteran population may limit generalizability.

Discussion

Prognostic Significance of Hyponatremia

Across the included studies, hyponatremia consistently emerged as an independent predictor of mortality in patients with cirrhosis, with this association observed across diverse clinical settings. Evidence from critically ill cohorts, such as that reported by Jenq et al. [12], demonstrated that low serum sodium retained prognostic significance even in patients with high baseline mortality. Similarly, studies in transplant waitlist populations, including those by Moini et al. [15], Londoño et al. [16], and Biggins et al. [17] confirmed its independent contribution alongside established scoring systems.

Importantly, in patients with lower MELD scores, investigations by Prohic et al. [13] and Heuman et al. [18] highlighted the ability of hyponatremia to identify individuals at increased risk of early death who may otherwise appear clinically stable. In advanced disease states, such as refractory ascites, Sersté et al. [14] demonstrated that severe hyponatremia remained a strong predictor of mortality. Taken together, these findings suggest that hyponatremia functions not merely as an isolated biochemical abnormality but rather as a systemic marker of circulatory and neurohumoral dysfunction, reflecting the underlying pathophysiological severity of liver disease across a broad clinical spectrum.

Pathophysiological Basis of Hyponatremia

From a pathophysiological perspective, the prognostic significance of hyponatremia in cirrhosis can be understood within the framework of progressive circulatory dysfunction. Portal hypertension leads to splanchnic vasodilation and a reduction in effective arterial blood volume, which in turn activates compensatory neurohumoral pathways, including the renin-angiotensin-aldosterone system and the non-osmotic release of vasopressin. This results in impaired free water excretion and dilutional hyponatremia, often accompanied by renal dysfunction and fluid accumulation [19].

The consistent association between low serum sodium and adverse outcomes across studies, including those by Jenq et al. [12], Sersté et al. [14], and Heuman et al. [18], supports the interpretation that hyponatremia is not simply correlated with mortality, but rather reflects an advanced stage of hemodynamic compromise. In this context, hyponatremia may represent the clinical manifestation of severe circulatory dysfunction, integrating portal hypertension, renal impairment, and neurohumoral activation into a single measurable parameter that captures the systemic derangements characteristic of decompensated cirrhosis [20].

Hyponatremia in Relation to the Model for End-Stage Liver Disease

The relationship between hyponatremia and established prognostic models provides important clinical insight into risk stratification in cirrhosis. Across the included studies, serum sodium consistently demonstrated independent prognostic value even after adjustment for MELD, as shown in cohorts by Moini et al. [15], Londoño et al. [16], and Biggins et al. [17]. Notably, in patients with lower MELD scores, studies by Prohic et al. [13] and Heuman et al. [18] highlighted that hyponatremia identifies individuals at increased risk of early mortality who may otherwise be classified as low risk based on MELD alone. This suggests that while MELD primarily reflects hepatic and renal dysfunction, serum sodium captures an additional dimension of circulatory and neurohumoral derangement. In this context, hyponatremia may complement MELD by providing insight into pathophysiological processes not fully represented by conventional laboratory indices, thereby enhancing the overall assessment of disease severity.

Subgroup-Specific Prognostic Patterns

The prognostic relevance of hyponatremia appears to vary across clinical subgroups while remaining consistently significant. In patients with low MELD scores, findings from Prohic et al. [13] and Heuman et al. [18] indicate that hyponatremia serves as a marker of elevated mortality risk despite relatively preserved laboratory parameters, underscoring its potential role in refining transplant prioritization. In advanced disease states, particularly among patients with refractory ascites, Sersté et al. [14] demonstrated that severe hyponatremia is strongly associated with mortality, likely reflecting profound circulatory dysfunction and systemic decompensation. Similarly, in critically ill populations, Jenq et al. [12] reported that hyponatremia retains prognostic significance even in the context of high baseline mortality, suggesting robustness across severity spectra. Collectively, these observations indicate that the prognostic value of hyponatremia is preserved across different stages of cirrhosis, although its relative contribution may vary depending on the underlying disease severity and clinical context.

Clinical Implications

The findings of this review have several implications for clinical practice, particularly in the domain of risk stratification in cirrhosis. Across multiple studies, including those by Moini et al. [15], Londoño et al. [16], and Biggins et al. [17], serum sodium demonstrated consistent prognostic value alongside established scoring systems, supporting its integration into composite models such as MELD-Na [21]. Additionally, the ability of hyponatremia to identify high-risk patients within ostensibly lower-risk groups, as shown by Prohic et al. [13] and Heuman et al. [18], highlights its potential utility in refining clinical decision-making and transplant prioritization. Monitoring serum sodium trends may also provide dynamic insight into disease progression and evolving hemodynamic status. However, these findings should be interpreted within the broader clinical context, as serum sodium represents one component of a multifactorial disease process rather than a standalone determinant of outcomes.

Methodological Considerations

The interpretation of these findings should be informed by several methodological considerations inherent to the included studies. All studies were observational in design, which introduces the potential for residual confounding despite the use of multivariable analyses, as seen in studies such as Jenq et al. [12] and Sersté et al. [14]. Furthermore, the included populations were heterogeneous, encompassing critically ill ICU patients, transplant waitlist cohorts, and individuals with advanced complications such as refractory ascites, which may influence both baseline risk and the strength of observed associations. Variability in the definition of hyponatremia across studies, ranging from mild reductions to severe thresholds, further complicates direct comparisons. These differences in study design, patient selection, and exposure definitions may contribute to variability in effect estimates and should be considered when interpreting the overall body of evidence [22,23].

Limitations of the Review

This systematic review has several limitations that should be acknowledged. First, the evidence base is predominantly derived from observational studies, with a lack of randomized data to establish causal relationships. Second, a quantitative meta-analysis was not performed, which limits the ability to generate pooled effect estimates and formally assess heterogeneity. Third, there was considerable variability in the definitions of hyponatremia, outcome measures, and follow-up durations across the included studies, which may affect comparability. In particular, the included studies used heterogeneous sodium thresholds and did not consistently report admission sodium levels, median sodium values, or sodium distributions among survivors and non-survivors. As a result, the prognostic impact of mild, moderate, and severe hyponatremia could not be compared directly across studies. Additionally, the inclusion of studies across diverse clinical settings introduces heterogeneity that may influence the generalizability of findings. Finally, the possibility of publication bias cannot be excluded, as studies reporting significant associations may be more likely to be published. Taken together, these factors underscore the need for cautious interpretation and highlight areas for future research.

Future Directions

Future research should aim to refine the prognostic utility of serum sodium by moving beyond single-time-point measurements toward a more dynamic assessment framework. While current evidence, including studies such as Londoño et al. [16] and Biggins et al. [17], largely relies on baseline sodium values at listing or admission, longitudinal trends in serum sodium may provide more nuanced insight into disease trajectory and evolving circulatory dysfunction. Evaluating temporal changes could help distinguish transient fluctuations from sustained derangements that carry greater prognostic significance. In addition, integration of serum sodium with emerging biomarkers reflecting renal injury and neurohumoral activation, as well as with hemodynamic parameters, may allow for a more comprehensive characterization of disease severity. These approaches could facilitate the development of more personalized and multidimensional risk prediction models that extend beyond existing frameworks such as MELD-Na, with the potential to improve prognostic precision while maintaining clinical applicability [24].

## Conclusions

Hyponatremia consistently emerges as a robust and independent predictor of mortality across a broad spectrum of patients with cirrhosis, including those in critical care settings, transplant waitlists, and advanced disease states such as refractory ascites. The available evidence suggests that serum sodium provides prognostic information that complements established models, such as the MELD, particularly by identifying high-risk individuals within lower MELD strata in whom risk may otherwise be underestimated. Mechanistically, hyponatremia reflects advanced circulatory and neurohumoral dysfunction, integrating key pathophysiological features of decompensated liver disease into a single clinically accessible parameter. Collectively, these findings support the incorporation of serum sodium into prognostic assessment frameworks while emphasizing that its interpretation should remain contextual and integrated within the broader clinical evaluation of cirrhosis. Further research is needed to refine its prognostic role through dynamic and multidimensional risk-prediction approaches.
